# The physio-biochemical characterization reflected different calcium utilization efficiency between the sensitive and tolerant peanut accessions under calcium deficiency

**DOI:** 10.3389/fpls.2023.1250064

**Published:** 2023-08-21

**Authors:** Kang Tang, Dengwang Liu, Na Liu, Ningbo Zeng, Jianguo Wang, Lin Li, Zinan Luo

**Affiliations:** ^1^College of Agriculture, Hunan Agricultural University, Changsha, Hunan, China; ^2^Arid Land Crop Research Institute, Hunan Agricultural University, Changsha, Hunan, China; ^3^Hunan Peanut Engineering & Technology Research Center, Changsha, Hunan, China; ^4^Shandong Academy of Agricultural Sciences, Jinan, Shandong, China

**Keywords:** peanut, calcium deficiency, calcium uptake and distribution, peanut yield, antioxidant enzymes

## Abstract

Peanut yield in southern China is usually limited by calcium deficiency in soil. Most previous studies have found that small-seed varieties showed higher tolerance than large-seed varieties (e.g. Virginia type) under calcium deficiency, however, our preliminary research found that sensitive varieties also existed in small-seed counterparts. Few studies have been conducted to characterize low-calcium tolerance among small-seed germplasms with genetic diversity, and the differences in physiological characteristics between sensitive and tolerant varieties has not been reported yet. Thus, in order to better understand such differences, the current study firstly collected and characterized a diversity germplasm panel consisting of 50 small-seed peanut genotypes *via* a 2-year field trial, followed by the physiological characterization in sensitive (HN032) and tolerant (HN035) peanut genotypes under calcium deficiency. As a result, the adverse effects brought by calcium deficiency on calcium uptake and distribution in HN032 was much larger than HN035. In details, calcium uptake in the aboveground part (leaves and stems) was reduced by 16.17% and 33.66%, while in the underground part (roots and pods), it was reduced by 13.69% and 68.09% under calcium deficiency for HN035 and HN032, respectively; The calcium distribution rate in the pods of HN035 was 2.74 times higher than HN032. The utilization efficiency of calcium in the pods of HN035 was 1.68 and 1.37 times than that of HN032 under calcium deficiency and sufficiency, respectively. In addition, under calcium deficiency conditions, the activities of antioxidant enzymes SOD, POD, and CAT, as well as the MDA content, were significantly increased in the leaves of HN032, peanut yield was significantly reduced by 22.75%. However, there were no significant changes in the activities of antioxidant enzymes, MDA content, and peanut yield in HN035. Therefore, higher calcium absorption and utilization efficiency may be the key factors maintaining peanut yield in calcium-deficient conditions for tolerant genotypes. This study lays a solid foundation for selecting low-calcium tolerant varieties in future peanut breeding.

## 1 Introduction

Peanut (*Arachis hypogaea* L.) is an important worldwide oilseed and economic crop. Soil acidification is getting intensified especially in the area with plenty rainfall and fertilization misuse, directly leading to calcium deficiency in the soil (Blair et al., 1988; Hue, 2004). The decrease of exchangeable calcium in acidic soil severely inhibits the absorption of calcium in peanut, thus affecting the growth and development of pods and impairing pod yield (Yang et al., 2020).

Calcium can improve the peanut tolerance in response to abiotic stresses. For example, the application of calcium fertilizer can alleviate damage to chloroplasts and cell nucleus caused by high temperature (Yang et al., 2015) and maintain the stability of plasma membrane (Zai et al., 2006) in peanut. Calcium application can also promote peanut growth, dry biomass accumulation, and photosynthetic capacity under cold stress at night (Song et al., 2020; Wu et al., 2020). In contrast, calcium deficiency significantly reduced the fresh biomass accumulation of shoot and root systems, and affected the expression of functional genes related to energy conversion, redox system, intracellular membrane, and cytoskeleton maintenance in peanut young embryos, potentially leading to yield loss and increased number of empty pods (Zhang et al., 2007; Li et al., 2014; Cui et al., 2019). Previous studies have shown that under low calcium conditions, the activities of catalase (CAT) and peroxidase (POD) in peanut leaves are significantly reduced, while the content of malondialdehyde (MDA), a lipid peroxidation product, is increased. This indicates that the active oxygen defense system in peanut plants is damaged, leading to a decrease in the rate of active oxygen elimination, accelerated plant aging, and the accumulation of a large amount of active oxygen. Ultimately, this results in damage to the cell plasma membrane, inhibition of protein synthesis, and adversely affects the healthy growth of peanut plants (Zhang et al., 2004).

Different peanut varieties confer different calcium demands. Large-seed varieties usually require higher calcium supplements than small-seed ones for optimal pod growth and development (Tillman et al., 2010). Gao et al. (2016) found that calcium deficiency in the early seedling stage did not affect the root and shoot growth in YZ9102 (small-to-middle-seed), while a significant reduction has been observed in the both shoot and root biomass in a large-seed variety (LH11). When compared to LH11, YZ9102 has shown a longer root system as well as higher calcium absorption as well as transportation capabilities. Smal et al. (1989a) also found that large-seed variety accumulated more calcium in the pod shell than small-seed one as the soil calcium levels increased. In a sand culture experiment, Zhao and Fang (2017) found that the efficiency of calcium utilization in different peanut varieties decreased under low calcium stress, but the changes in calcium production efficiency and dry matter production efficiency varied among the varieties. Chen (2018) found that the different peanut varieties have varying sensitivities to low calcium conditions mainly due to their different abilities to absorb/accumulate Ca^2+^ in the seeds. Under calcium deficiency, the Ca^2+^ content in the fruit pegs, pods, and seeds of low-calcium sensitive varieties was significantly lower than that under calcium sufficiency, especially in the seeds and pods. However, no significant difference was observed in the seeds of calcium-tolerant varieties.

However, our preliminary studies found that large variation also existed for calcium demands in small-seed peanut varieties. The majority of current researches in peanut abiotic stress mainly focused on salt, cold, drought, and waterlogging resistance (Liu et al., 2015; Liu et al., 2017; Patel et al., 2022; Zeng et al., 2022), with no research conducted to identify and select small-seed varieties conferring low-calcium tolerance. Therefore, the current study aims to explore and select two peanut accessions with different low-calcium tolerance, followed by the physio-biochemical characterization in the two accessions. By comparing the physio- biochemical differences between the two accessions, the current study will lay a solid foundation for future low-calcium tolerance breeding in the peanuts.

## 2 Materials and methods

### 2.1 Experimental design

#### 2.1.1 Field screening experiment

A total of 50 small-seed peanut accessions introduced from abroad were used for the 2019-2020 field trials (**Table S1**), The field trials in 2019-2020 were conducted in Shaoyang (N23.08°, E111.59°) and Yongzhou (N25.37°, E112.18°) in Hunan province with an average annual temperature of 17.4°C and 18.9°C, annual precipitation of 1484.2 mm and 1403 mm, and daylight length of 1327 h and 1224 h, respectively. The soil types at two sites are typically acidic soil lacking of exchange able calcium with calcium levels lower than 800 mg.kg^-1^ (He, 1994; Wang et al., 2020), as detailed in **Table 1**. The accessions were sown on May 30, 2019, and June 1, 2020, and harvested on September 31, 2019, and October 1, 2020, respectively. A 2-year field trial was conducted in a randomized complete block design (RCBD) with two calcium treatment levels. The calcium treatment applied base lime (1125 kg.hm^-2^) before sowing, and the calcium deficiency treatment applied no base lime. The experimental plot (2.5 m × 0.8 m) was ridge-planted with two rows (ridge width 0.5 m and furrow width 0.3 m). The row spacing on the same ridge was 30 cm, and the sowing density of intra-row plants was 10 cm. The experimental plot was applied 750 kg.hm^-2^ compound fertilizer (N 15%, phosphorus oxide (P_2_O_5_) 15%, and potassium oxide (K_2_O) 15%) as basal fertilizer. All the experimental treatments were replicated 3 times.

**Table 1:** Physical and chemical properties of soil in the test site.

Site	pH (H_2_O)	OM (g.kg^-1^)	N (mg.kg^-1^)	P (mg.kg^-1^)	K (mg.kg^-1^)	Ca (mg.kg^-1^)
Shaoyang	5.05	7.75	49.00	0.80	61.00	600.00
Yongzhou	5.10	NA	122.00	0.40	74.00	316.00
Miluo	5.15	18.60	96.00	2.90	97.00	750.00

0-20 cm tillage layer. OM stand for organic matter; N stand for hydrolysis of sex of nitrogen; P, available phosphate; K, rapidly available potassium; Ca, exchangeable calcium; NA, Not Available.

#### 2.1.2 Field trial of two different genotypes for calcium tolerance

The field trial in 2021 was conducted in Yueyang, Hunan, China (N28°55′53.17, E113°09′49.95), with an average annual temperature of 17.0°C, precipitation of 1345 mm, and daylight length of 1650 h. The soil types are typically acidic soil lacking of exchange able calcium with calcium levels lower than 800 mg.kg^-1^ (He, 1994; Wang et al., 2020), as detailed in **Table 1**. The two selected accessions were sown on May 1, 2021, and harvested on September 1, 2021. The 2021 trial was also set up with two calcium treatments. The calcium treatment applied base lime (750 kg.hm^-2^) before sowing, and the calcium deficiency treatment applied no base lime. A total of 2000 seeds of two selected accessions HN0035 (low-calcium high tolerance, HT) and HN0032 (low-calcium low tolerance, LT) were planted with a plot size of 80 square meters. Each plot was replicated 3 times. The sowing density, fertilization management as well as pest, insect and weed control management were consistent with the field trials in 2019-2020.

### 2.2 Phenotypic evaluation

#### 2.2.1 Relative chlorophyll content and photosynthetic parameters

A hand-held soil–plant analysis development chlorophyll meter (SPAD–502) (Minolta, Osaka, Japan) was used to determine the relative chlorophyll content (SPAD values). The net photosynthetic rate (Pn), stomatal conductance (Gs), intercellular CO_2_ concentration (Ci), and transpiration rate (Tr) were measured using a Li–6400XT portable photosynthesis device (Li–COR, Lincoln, NE, USA). SPAD values and photosynthetic parameters of the third leaves from the main stem were measured between 9:00 and 11:00 AM on a sunny day.

#### 2.2.2 The activities of antioxidant enzymes (SOD, POD, CAT) and MDA contents

The determination of POD, SOD, and CAT activities was carried out using the guaiacol colorimetric method, NBT photoreduction method, and UV absorption method, respectively (Chance, 1955; de Souza et al., 2021). The MDA content was determined using the thiobarbituric acid method (Lin et al., 1984). The specific experimental procedure was carried out according to the study conducted by Li et al. (2023).

#### 2.2.3 Plant architecture and dry biomass accumulation

Seedling emergence was investigated from 10 days after sowing until the maximum number of seedlings was reached and the emergence rate was calculated. At harvest time, the number of plants harvested from the plots was counted to calculate Percentage growing into plants. Six representative plants at the same growing stage were collected from each plot at the flower-pegging, pod-setting, and pod-filling stage, respectively. Three out of six plants were used to measure main stem height (cm), lateral branch length (cm) and the branch number. After measurement, six plants were split into roots, stems, leaves and pods (including pegs) into paper bags and dried at 105°C for 30 min, followed by 80°C for 48 h until complete dehydration. The dry biomass of each organ was then weighted, and the dry matter collected from pod-setting and pod-filling stages were crushed and saved for later elemental determination.


(1) Root to crown ratio (R/S) = root dry weight/ above−ground biomass

(2) Vegetative organs/reproductive organs (V/R) = Dry weight of nutrient organs / Dry weight of reproductive organs

(3) Economic index =Pod yield / Total plant dry weight

#### 2.2.4 The determination of yield and its components

The pods from ten representative plants were harvested from each plot at maturity stage, followed by sunlight dehydration for weight determination. The number of full pods, number of unsaturated pods, number of empty pods, number of total pods, dry biomass of pod shells, pod length, pod width, weight of full and unsaturated pods and seeds were recorded. Calculation of yield per plant, hundred pods Weight, hundred seeds Weight, number of kilograms of pods, kernel rate, full pod weight percentage, full seed weight percentage, empty pod rate and plumpness degree of pod.

#### 2.2.5 Calcium content determination

Calcium element was determined by graphite furnace ablation-inductively coupled plasma mass spectrometry. The crushed powder was weighed to 0.5 g plus 5 ml of nitric acid in a digestion tube with a bent-neck funnel overnight. The powder was digested in a graphite furnace the next day until the solution in the tube was nearly clear and transparent. After cooling, the solution was transferred to a 50 ml volumetric flask with fixed volume and mixed, filtered through a microporous membrane in a 10 ml centrifuge tube, and the Ca content was determined in an inductively coupled plasma mass spectrometer (Model: ICPE-9000). The accumulation of calcium element in each organ was measured using the following formats:


(4) Calcium uptake (mg per plant) = Calcium content * dry matter mass

(5) Calcium partitioning rate (%) = Calcium uptake in each organ / calcium uptake of the whole plant * 100%

(6) $$\text{Calcium}\;\text{saponin}\;\text{production}\;\text{efficiency}\;\left(\text{kg}.\text{k}{\text{g}}^{-1}\right)\;=\;\text{pod}\;\text{yield}\;/\;\text{total}\;\text{accumulated}\;\text{calcium}\;\text{saponins}\;\text{per}\;\text{plant}$$

### 2.3 Statistical analysis

#### 2.3.1 Calculation of comprehensive evaluation value (D)

The low-calcium tolerance coefficient (Rx) of 33 phenotypic traits was evaluated for principle component analysis (PCA) following the formula (7); the characteristic value *α_ij_* obtained from PCA was used to calculate membership function value *X_j_* following the formula (8); the *X_j_* was then used to calculate membership function value *U(X_j_)* with the formula (9); the comprehensive evaluation *D*-value was used to evaluate the low-calcium tolerance for each peanut genotype based on the formula (10) and (11).


(7) Rx= phenotypic value under calcium deficiency/ under sufficient calcium

(8) $${\text{X}}_{\text{j}}=\sum_{\text{i}=0}^{\text{n}}{\text{a}}_{\text{ij}}{\text{X}}_{\text{ij}}$$

(9) $$\text{U}\left({\text{X}}_{\text{j}}\right)=\left({\text{X}}_{\text{j}}-{\text{X}}_{\text{j},\text{min}}\right)/\left({\text{X}}_{\text{j},\text{max}}-{\text{Xj}}_{\text{j},\text{min}}\right)$$

*α_ij_* stands for the eigenvector that is related to eigenvalue of Rx for each phenotypic trait; *X_ij_* is the standardized Rx of each trait; *X_j_* represents the jth comprehensive index; Xx,max and Xx,min represent the maximum and minimum values of the *jth* comprehensive index.


(10) $$W_{j}=\frac{P_{j}}{\sum_{j=1}^{n}P_{j}}$$

(11) $$D=\sum_{j=1}^{n}(U(X_{j})xW_{j})$$

*W_j_* stands for the weight of the *jth* comprehensive index, and *P_j_* represents the variance contribution rate of the *jth* comprehensive index.

Systematic clustering was conducted according to the above obtained *D*-value using Euclidean distance square method at the Euclidean distance of 5 (Zhang et al., 2013).

#### 2.3.2 Statistical analysis of two extreme peanut accessions

Data were analyzed using the software IBM SPSS v21.0. The one-way Analysis of Variance (ANOVA) with Duncan’s test was used to characterize the differences between different genotypes under two calcium treatments. Differences were considered statistically significant at p <0.05. All the relevant figures were generated by OriginPro 2019.

## 3 Results

### 3.1 Selection and evaluation of low-calcium tolerant genotypes from small-seed peanut germplasm panel

**Figure 1:**
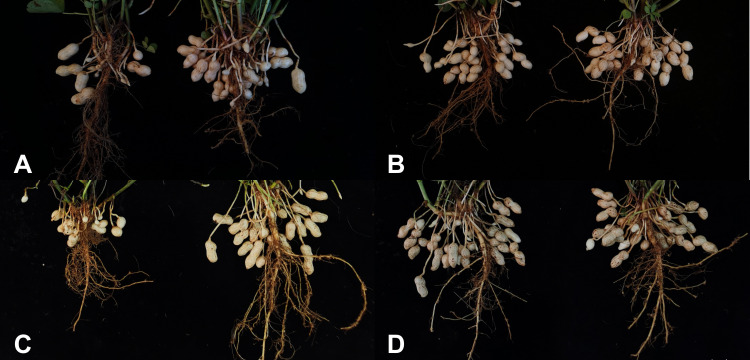
The pod morphological differences in two accessions under different calcium treatments. **(A)** the pod morphology in “LT” under different calcium treatments at pod-setting stage; **(B)** the pod morphology in “HT” under different calcium treatments at pod-setting stage; **(C)** the pod morphology in “LT” under different calcium treatments at pod-filling stage; **(D)** the pod morphology in “HT” under different calcium treatments at pod-filling stage.

**Figure 2:**
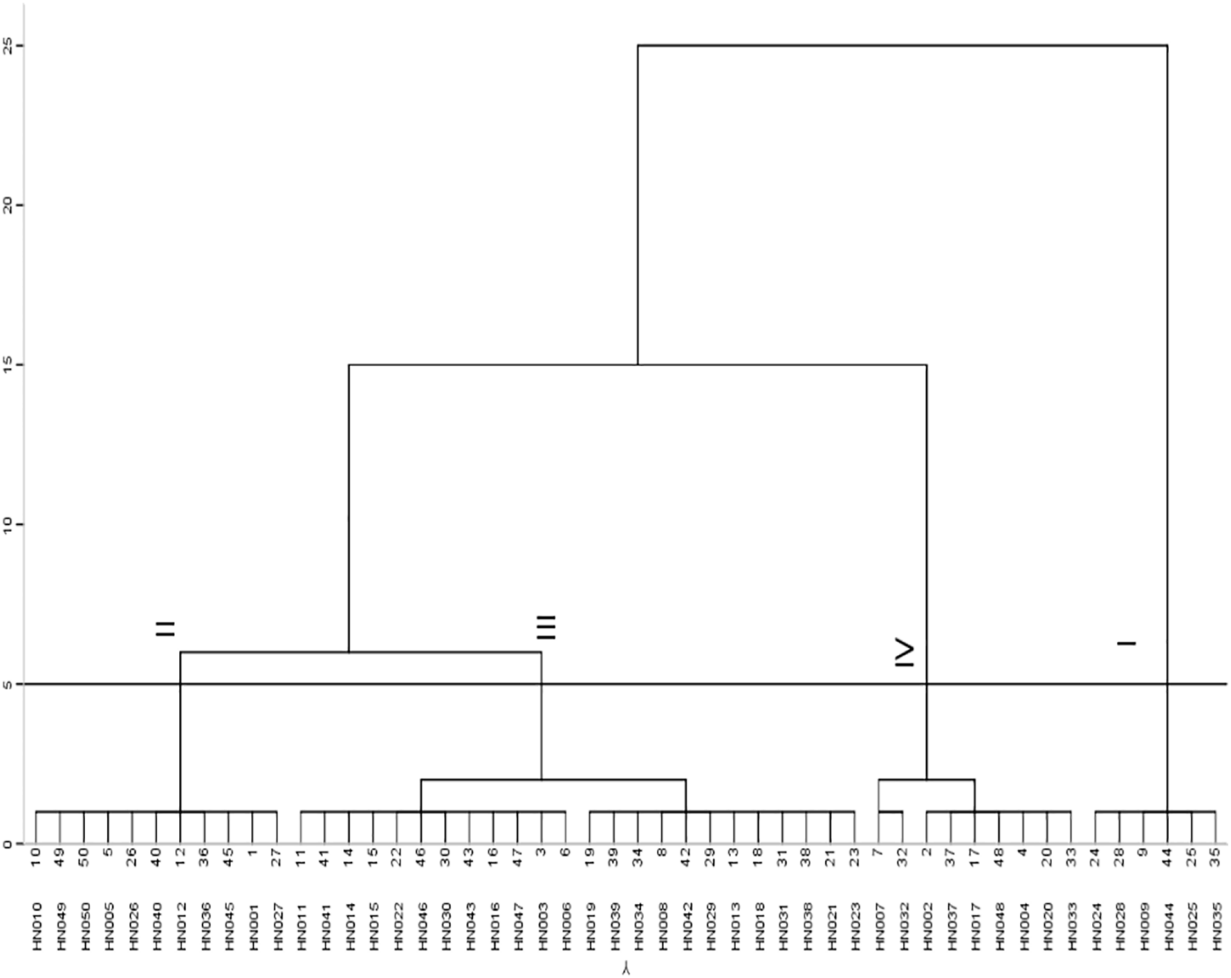
Cluster diagram of D values of 50 peanut accessions. I: strong tolerance; II: considerable tolerance; III: intermediate type; IV: calcium sensitivity.

A PCA of the low-calcium tolerance coefficients calculated from 33 phenotypic traits revealed that the cumulative contribution of the first 10 principle components (PC) reached 80.088%, explaining most of the phenotypic variation (**Table S2**). The most weighted traits in the first PC were yield and its components including yield (0.878), number of filled pods per plant (0.759), pod plumpness (0.777), pod shell (0.756), and seed kernel (0.867); Quality traits including oleic acid (0.616), linoleic acid (-0.682), and oleic to linoleic acid ratio (0.681) showed highest weight in the second PC; Seedling growth ability suggested highest weight among the third PC (**Table S2**). The comprehensive evaluation value (*D*) showed that HN035 ranked the highest, with has the *D* value of 0.598, suggesting strongest low-calcium tolerance, while HN032 performing smallest *D*-value of 0.297 indicated the most weakness in low-calcium tolerance (**Figure 1**, **Table S3**). The systematic clustering suggested that the 50 peanut accessions can be classified into four categories ranging from the lowest to highest low-calcium tolerance (**Figure 2**). Six accessions with highest low-calcium tolerance included HN024, HN028, HN009, HN044, HN025, HN035, while nine accessions including HN002, HN037, HN033, HN020, HN048, HN017, HN004, HN007 and HN032 conferred the lowest low-calcium tolerance (**Figure 2**). Hence, HN035 (HT) and HN032 (LT) were selected for the subsequent analyses.

### 3.2 The effects of calcium deficiency on photosynthetic characteristics, antioxidant enzyme activity, plant architecture, biomass accumulation, yield and its components in two different accessions

#### 3.2.1 The effects of calcium deficiency on photosynthetic characteristics in different peanut accessions

**Figure 3:**
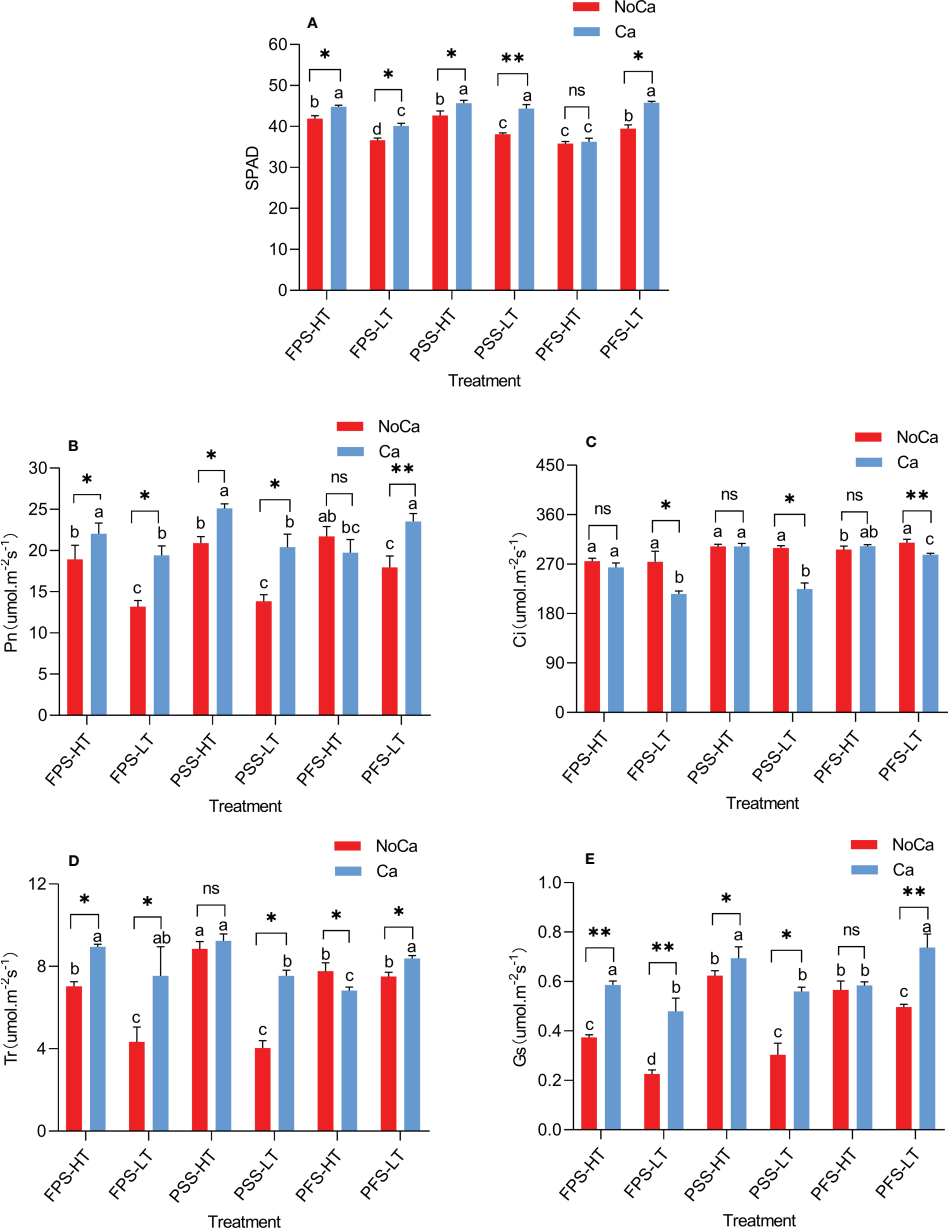
SPAD and photosynthetic characteristics at different stages in different peanut accessions under different calcium treatments. **(A)** Chlorophyll SPAD values of two accessions at different stages under different calcium treatments. **(B)** Photosynthetic rates of two accessions at different stages under different calcium treatments. **(C)** Carbon dioxide concentration at saturation of two accessions at different stages under different calcium treatments. **(D)** Transpiration rates of two accessions at different stages under different calcium treatments. **(E)** Stomatal conductance of two accessions at different stages under different calcium treatments. FPS: flower-pegging stage; PSS: pod-setting stage; PFS: pod-filling stage; HT represents low-calcium high tolerance, and LT represents low-calcium low tolerance; NoCa, 0 kg.hm^-2^; Ca,750 kg.hm^-2^. “Pn” Net Photosynthetic rate; “Gs” Stomatal conductance; “Tr” transpiration rate; “Ci” intercellular CO_2_ concentration. Different lowercase letters represent significant (P<0.05). “*” represents p< 0.05, “**” represents p< 0.01, "ns" non-significant.

Significant differences were observed in two accessions in terms of chlorophyll SPAD value, Pn, Gs, Tr, and Ci during three growing stages (**Figure 3**). During the full fruit stage, the SPAD value in HN035 did not change significantly, while a significant decrease of 13.70% was observed in HN032 under calcium deficiency (**Figure 3A**). The Pn of both accessions were adversely affected until the PFS stage, where no significant change was observed in HN035, whereas a significant decrease of 23.63%in HN032 was shown in **Figure 3B**. The change of Ci in HN035 was not significant during all the three growing stages, but significant increases ranging from 7.70% to 33.30% were observed in HN032 across the three growing periods under calcium deficiency (**Figure 3C**). The Tr and Gs in HN032 were significantly inhibited under calcium deficiency during all the three periods, but the capability of Tr and Gs in HN035 were recovered during pod-setting stage and full fruit stage, respectively (**Figures 3D, E**). The tolerance accessions were not affected by low calcium of Tr at pod-setting and Gs at pod-filling stage and the difference was not significant. The change of intercellular carbon dioxide concentration in tolerant accessions was not significant in each period, but the change was great in sensitive accessions, which increased significantly in the three periods, with the increase of 33.30% in full fruit stage under calcium deficiency.

#### 3.2.2 The effects of calcium deficiency on the activities of antioxidant enzymes and MDA content vary among different peanut accessions

**Figure 4:**
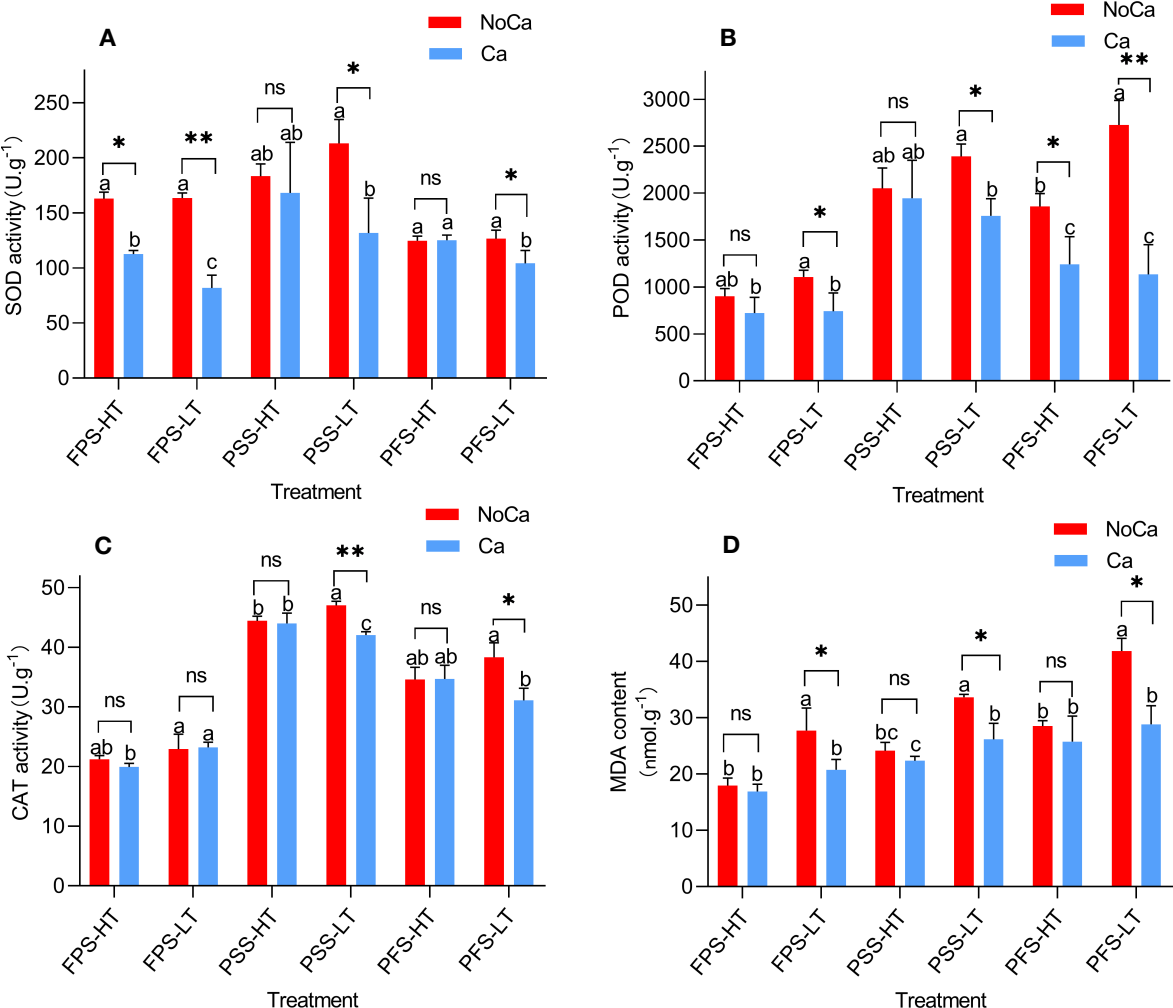
*Antioxidant enzyme activities SOD, POD, CAT, and MDA contents* at different stages in different peanut accessions under different calcium treatments. **(A)** SOD activities of two accessions at different stages under different calcium treatments. **(B)** POD activities of two accessions at different stages under different calcium treatments. **(C)** CAT activities of two accessions at different stages under different calcium treatments. **(D)** MDA contents of two accessions at different stages under different calcium treatments. FPS: flower-pegging stage; PSS: pod-setting stage; PFS: pod-filling stage; HT represents low-calcium high tolerance, and LT represents low-calcium low tolerance; NoCa, 0 kg.hm^-2^; Ca,750 kg.hm^-2^.Different lowercase letters represent significant (P<0.05). “*”represents p< 0.05, “**”represents p< 0.01, "ns" non-significant.

The activities of antioxidant enzymes and the levels of MDA content in the leaves of the two accessions increased to varying degrees under calcium deficiency (**Figure 4**). The activities of SOD and POD in HN032 showed significant increases at three stages under calcium deficiency, while HN035 only showed significant changes during the flower-pegging stage (**Figures 4A, B**). HN032 showed minor changes in CAT activity during the early stage under low calcium conditions, but significant changes were observed during the pod and ripe fruit stages. HN035 showed no significant changes in CAT activity (**Figure 4C**). The MDA content of HN032 showed a significant increase of 28.59-45.13%, while HN035 showed no significant change under calcium deficiency (**Figure 4D**).

#### 3.2.3 The difference in plant architecture of two accessions under calcium deficiency

The main stem height, lateral branch length, shape index and number of branches were measured after harvest. Significant differences were only observed in HN032 with a decrease by 11.75% and 9.60% in main stem height and lateral branch length under calcium deficiency, respectively (**Table 2**).

**Table 2:** Plant traits of two types of accessions at maturity period.

Type	Treatment	MSH (cm)	LBL (cm)	NB	SI
HT	NoCa	54.27 ± 0.85b	56.77 ± 2.17b	7.00 ± 0.34a	1.05 ± 0.010a
	Ca	56.03 ± 1.36b	58.53 ± 1.19b	7.55 ± 1.35a	1.04 ± 0.011a
LT	NoCa	56.10 ± 1.67b	58.60 ± 1.08b	7.33 ± 1.15a	1.04 ± 0.01a
	Ca	63.57 ± 2.69a	66.37 ± 0.85a	7.44 ± 0.70a	1.04 ± 0.01a

HT represents low-calcium high tolerance, and LT represents low-calcium low tolerance; NoCa, 0 kg.hm^-2^; Ca, 750 kg.hm^-2^. MSH, main stem height; LBL, lateral branch length; NB, Number of branches; SI, Shape Index. Different lowercase letters represent significant (P<0.05).

#### 3.2.4 The effects of calcium deficiency on dry matter accumulation in different peanut accessions

Even though the dry matter accumulation of vegetative organs significantly changed in both accessions, a significant decrease of 61.28% was only observed in the pods (reproductive organ) of HN032 (**Table 3**). In addition, the V/R ratio significantly increased 97.36% and economic index significantly dropped with 43.04%, respectively. However, no significant change was observed in pod biomass, V/R and the economic index in HN035 (**Table 3**).

**Table 3:** Dry matter accumulation of each organ in two accessions under different calcium treatments.

Type	Treatment	Root (g)	Stem (g)	Leaf (g)	Pod (g)	Vegetative organ (g)	R/S	V/R	Economic index
HT	NoCa	0.54 ± 0.04b	12.86 ± 0.74b	13.45 ± 0.47a	12.46 ± 1.44a	26.85 ± 0.87c	0.021ab	2.18 ± 0.28b	0.316 ± 0.03a
	Ca	0.66 ± 0.02a	13.17 ± 0.49b	14.36 ± 0.51a	13.06 ± 2.12a	28.19 ± 0.64b	0.024a	2.20 ± 0.40b	0.316 ± 0.04a
LT	NoCa	0.43 ± 0.04c	11.85 ± 0.73b	11.46 ± 0.79b	3.57 ± 0.44b	23.74 ± 0.1d	0.018bc	6.73 ± 0.88a	0.131 ± 0.01c
	Ca	0.48 ± 0.04bc	15.87 ± 1.26a	14.49 ± 0.58a	9.22 ± 1.58c	30.84 ± 0.79a	0.016c	3.41 ± 0.60b	0.230 ± 0.03b

HT represents low-calcium high tolerance, and LT represents low-calcium low tolerance; NoCa, 0 kg.hm^-2^; Ca, 750 kg.hm^-2^. R/S represents root dry weight/above-ground biomass; V/R represents ratio of vegetative body to reproductive body. Different lowercase letters represent significant (P<0.05).

#### 3.2.5 The effects of calcium deficiency on the yield and composition of different peanut varieties

As for yield and its components, significant changes were observed in all the collected phenotypic traits in HN032 while no significant change in HN035 (**Table 4**). Especially, the rate of empty pods was largely increased with 182.80% and yield per plant was dropped with 18.37% in HN032 (**Table 4**).

**Table 4:** The differences in yield and its components in two accessions under different calcium treatments.

Type	Treatment	NFP	NEP	YP (g.plant^-1^)	EPR (%)	HPW (g)	HSW (g)	KR (%)	NKP
HT	NoCa	17.31 ± 1.13a	1.23 ± 0.25b	18.69 ± 1.43a	6.02 ± 1.24b	104.73 ± 2.91a	43.67 ± 1.22a	68.14 ± 0.97b	1014.77 ± 41.78c
	Ca	17.17 ± 0.85a	1.10 ± 0.30b	19.44 ± 1.74a	5.49 ± 2.02b	110.15 ± 3.67a	44.99 ± 1.22a	68.96 ± 4.64b	963.62 ± 18.17c
LT	NoCa	13.13 ± 0.64b	2.73 ± 0.12a	9.71 ± 0.28c	15.13 ± 1.01a	66.32 ± 1.43c	35.76 ± 1.04c	70.37 ± 1.02b	1572.26 ± 33.83a
	Ca	16.27 ± 1.21a	1.00 ± 0.20b	12.57 ± 0.26b	5.35 ± 0.67b	74.16 ± 4.16b	38.93 ± 0.53b	78.48 ± 3.07a	1408.64 ± 70.84b

HT represents low-calcium high tolerance, and LT represents low-calcium low tolerance; NoCa, 0 kg.hm^-2^; Ca, 750 kg.hm^-2^. NFP Number of full pods; NEP Number of empty pods; EPR Empty pod rate;YP Yield per plant; HPW Hundred pods Weight; HSW Hundred seeds Weight; KR Kernel rate; NKP Number of kilograms of pods. Different lowercase letters represent significant (P<0.05).

### 3.3 The effects of calcium deficiency on calcium uptake, distribution, and utilization efficiency in different peanut accessions.

**Figure 5:**
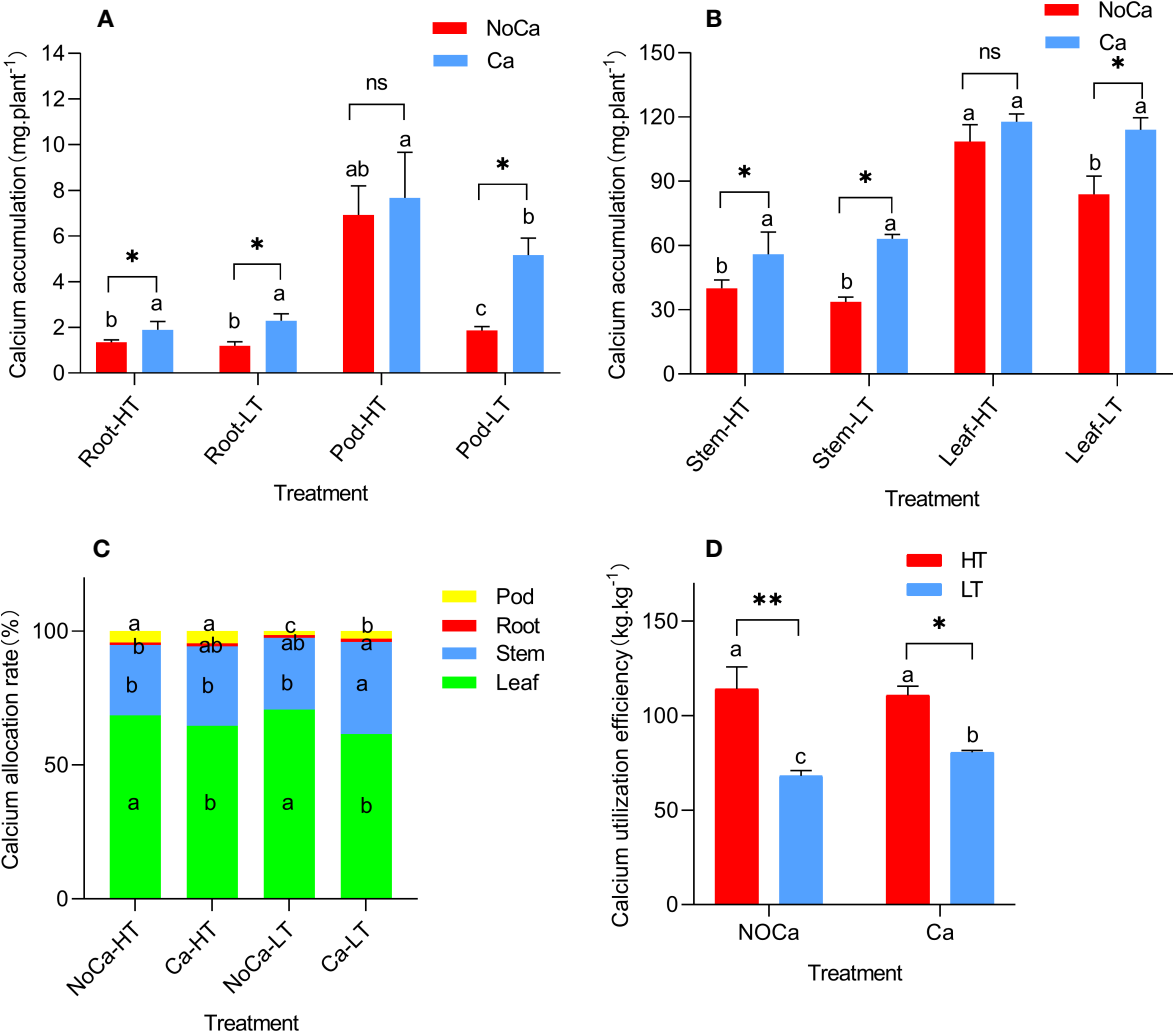
Calcium absorption, distribution and utilization efficiency in different accessions. **(A)** Calcium accumulation in underground parts. **(B)** Calcium accumulation in aboveground parts. **(C)** Calcium distribution rate in each organ. **(D)** Calcium utilization efficiency. HT represents low-calcium high tolerance, and LT represents low; NoCa, 0 kg.hm^-2^; Ca,750 kg.hm^-2^. Different lowercase letters represent significant (P<0.05). “*”represents p< 0.05, “**”represents p< 0.01, "ns" non-significant. -calcium low tolerance.

In general, the calcium in the aboveground and underground organs were mainly accumulated in leaves and pods, respectively (**Figure 5**). Especially, HN035 accumulated 3.70 and 1.49 times more calcium than HN032 in the pods under calcium deficiency and sufficiency (**Figure 4A**). The calcium distribution rate of HN035 reached 2.74 and 1.65 times higher than HN032 in the pods under calcium deficiency and sufficiency (**Figure 5C**). The utilization efficiency of calcium in the pods of HN035 was 1.68 and 1.37 times than that of HN032 under calcium deficiency and sufficiency, respectively (**Figure 5D**).

## 4 Discussion

### 4.1 Germplasm screening identified two accessions with different tolerance under calcium deficiency

Soil calcium deficiency adversely affects peanut yield, and the selection of low-calcium tolerant varieties is one of the most effective way to realize high-yield peanut breeding under calcium deficiency. (Wang et al., 2022) classified 32 peanut accessions into three clusters based on their pod yield and the ratio of unfulfilled pods under calcium deficiency. Among the low-calcium sensitive accessions, two small-seed cultivars were observed, indicating the tolerance variation in small-seed accessions. Likewise, in our previous research, relative yield (the average yield of low-calcium peanut for a certain variety/the average yield of low-calcium peanut for all the participating varieties) and the tolerance coefficient to low-calcium of yield were used for clustering analysis for 105 peanut accessions with various seed size from 15 provinces in China, resulting in five clustered categories: extremely strong (1.90%), strong (0.95%), moderate (18.10%), sensitive (78.10%, 39 large-seed, 5 medium-seed, and 8 small-seed varieties), and highly sensitive (0.95%, one large-size) under calcium deficiency. We found that the majority of sensitive materials were accounted for by medium and large peanut accessions, but a few small-seed sensitive accessions also exist (Kang et al., 2021). Thus, we further focused on screening tolerant accessions among small-seed peanut germplasm.

The PCA based on a total of 33 indexes of low-calcium tolerance involving plant architecture, chlorophyll content, yield and its components, quality, and calcium content was applied to reflect different weight in various phenotypic traits. The clustering analysis based on *D*-value divided the current germplasm into four categories: strong tolerance (12%),considerable tolerance (22%), intermediate type (48%), and calcium sensitivity (18%), showing that the vast majority of small-seeded accessions were not sensitive to low-calcium stress, but variation in sensitivity still can be observed in accordance with previous studies (Kang et al., 2021; Wang et al., 2022). Among the two extreme categories, HN035 and HN032 were selected for further analysis (**Figure 1**). The germination rate, the number of full pods per plant, yield per plant, and calcium content of the kernels decreased under calcium deficiency in HN032, while no changes were observed in HN035 (**Table S4**).

### 4.2 Low-calcium tolerant accession conferred higher photosynthesis capability than the sensitive one

Photosynthesis is an essential process for plant growth and development. When plants are faced with environmental stress, free calcium ions (Ca^2+^) are immediately released from calcium repositories and can serve as second messengers to stimulate downstream calcium-dependent metabolic (signaling transduction) pathways. Free Ca^2+^ could also couple with calcium- modulating proteins such as calmodulin (CaM) and calcium-binding proteins (CAS) to mediate stomatal movement, hydrolysis reactions, photosynthetic electron transfer (Kukuczka et al., 2014; Wang et al., 2019; Park and Shin, 2022), photosynthetic carbon assimilation (Kreimer et al., 1988), and photoprotection activities (Maciej et al., 2017). In the current study, HN035 conferred higher SPAD value, photosynthetic rate, stomatal conductance, and transpiration rate in all three periods compared to the sensitive one (HN032), indicating stronger photosynthetic capability in the tolerant accession than the sensitive one under calcium deficiency (**Figures 3A, B, D, E**). This coincidently matches a previous study related to salt stress in wild soybean, which suggested a higher net photosynthetic rate (Pn) in the salt-tolerant accession than the sensitive one under salt stress (Jiao et al., 2018). It is worth noting that the intercellular CO_2_ concentration in HN032 increased significantly in all the three periods under stress, while no significant change was observed in HN035 (**Figure 3C**). Calcium application could improve photosynthesis capability for both accessions *via* increasing SPAD value, Pn, Tr, Gs and reducing Ci (**Figure 3**). In accordance with our study, previous studies also found that photosynthesis of the sensitive variety was greatly affected by calcium deficiency and that calcium supplementation can enhance photosynthesis capability (He et al., 2018; Duan et al., 2020; Hu et al., 2022).

### 4.3 The changes of antioxidant enzymatic activities in the sensitive accession under calcium deficiency were more pronounced than the tolerant one

The antioxidant enzymes play a crucial role in protecting plants from oxidative stress under both abiotic and biotic stresses (Comadira et al., 2015). A significant increase was observed in the activities of SOD, PDD, and CAT in the low-calcium sensitive accesss (**Figures 4A-C**), which was consistent with the findings of previous studies in peanut and tobacco under nitrogen deficiency stress (Rubio-Wilhelmi et al., 2011; Patel et al., 2020). In contrast, the antioxidant enzymatic activities in tolerant accession remained unchanged, indicating that the degree of oxidative damage to tolerant varieties was mild when compared to sensitive accessions. A study conducted on TG26 also found that SOD and CAT levels remained unchanged under phosphorus or nitrogen deficiency conditions, suggesting that SOD and CAT might be lower than the threshold levels for scavenging excessive production of O_2_^-^ as well as maintaining hydrogen peroxide levels (Panda et al., 2019). The content of MDA can be used to evaluate the extent of oxidative damage caused by abiotic stress in plants. Researchers have studied different salt-tolerant, drought-tolerant, and cold-tolerant peanut varieties and found that the MDA content in sensitive varieties showed greater increases than the tolerant ones. This study indicated that the MDA content increased significantly in the sensitive accession HN032 as the growth period progressed under low calcium stress (**Figure 4D**). Tolerant accessions, on the other hand, showed no significant changes in MDA content across the three growth stages and accumulated a much lower amount of MDA when compared to the sensitive accession, suggesting that they experienced less oxidation damage to their cell membranes and generated less MDA through lipid peroxidation. These results were consistent with previous studies.

### 4.4 Low-calcium tolerant accession performed superiority in the agronomic characteristics than the sensitive one

This current study has found significant changes in plant architecture and dry biomass accumulation in the sensitive accession (HN032), while little effects have been observed in HN035 with stronger tolerance under calcium deficiency (**Table 3**). These findings showed consistency with previous studies under abiotic stress. For example, Liu et al. (2019) found in a hydroponic experiment that large amount of methylesterified pectin was formed under calcium deficiency, coupled with cell wall degradation, inhibition of root elongation and lateral root development, leading to decreased biomass and root growth in peanut. Compared to calcium sufficiency, calcium deficiency led to a significant decrease in dry biomass of nutritional and reproductive organs in HN032, while little change was observed in HN035 (**Table 3**), indicating that calcium deficiency would lead to growth redundancy in the vegetative development in the sensitive accession (HN032), thus possibly break the balance between vegetative and reproductive growth and impede the normal transportation of photosynthetic products from vegetative or above-ground organs to reproductive organs.

As for yield and its components, the sensitive accession (HN032) showed large reduction under calcium deficiency when compared with HN035 (**Table 4**), which did not show any significant effects on yield per plant, number of full pods per plant, 100-seed weight, 100-kernel weight, and empty pod ratio. However, significant reductions in yield and the number of full pods per plant were observed in HN032, with a rough two-time increase observed in the empty pod ratio (**Table 4**). This is consistent with previous researches (Aniefiok et al., 2021; Kadirimangalam et al., 2022; Yang et al., 2022), where tolerant peanut accession have shown stable yields under calcium deficiency due to their higher number of full pods per plant and small number of empty pods per plant.

### 4.5 Low-calcium tolerant accession conferred higher calcium utilization efficiency (CaUE) than the sensitive one

Peanuts require large amount of calcium for pod fulfillness. However, calcium ions are difficult to be transported from vegetative organs to pods in peanuts (Skelton and Shear, 1971), thus the calcium needed for pod development must be absorbed from the surrounding soil (Smal et al., 1989a; Zharare et al., 2012). Different peanut varieties showed various demand for calcium nutrition, e.g.Virginia-type (large-seed) peanuts required more calcium (Ca) than Spanish-type (small-seed) peanuts to achieve high yield (Smal et al., 1989b; Zharare et al., 2012). Moreover, different genotypes showed different calcium absorption, transportation, and utilization under calcium deficiency (Romheld and Marschner, 1986). For example, Gao et al. (2016) found that peanut seedlings of YZ9102 have higher calcium absorption and transportation abilities than LH11 under low calcium treatment during the seedling stage.

In agreement, the current study found that calcium accumulation in the leaves and pods of HN035 were higher than HN032 (**Figure 5A**, **4B**) under calcium-deficiency. Even though calcium allocation in leaves were significantly increased in both HN035 and HN032, no significant changes were observed in roots, stems and pods in HN035 (**Figure 5C**) while a significant decrease was identified in HN032, indicating the difficulty in calcium transportation from vegetative to reproductive organs in the sensitive peanut accession (Behling et al., 1989). In general, the CaUE of HN035 (114.11kg.kg^-1^, 110.70kg.kg^-1^) was significantly higher than HN032 (68.06kg.kg^-1^, 80.53kg.kg^-1^) under calcium deficiency and sufficiency, but no significant change was observed in HN035 under different calcium treatments, while CaUE in HN032 was significantly impeded under calcium deficiency (**Figure 5D**), The higher CaUE in HN035 may be due to stronger calcium absorption capability and distribution efficiency. Overally speaking, the differences in low-calcium tolerance were possibly due to different CaUE in two accessions.

## Conclusion

This study conducted a two-year field trial to screen 50 small-seed peanut accessions for their low-calcium tolerance and select two accessions with significant differences in low-calcium tolerance. The physio-biochemical studies found that the tolerant peanut accession HN035 showed mild degree of cellular oxidative damage, stronger photosynthetic performance, higher number of full pods per plant, and higher calcium utilization efficiency (CaUE) under calcium deficiency than the sensitive accession HN032. This study will provide a good foundation for subsequent breeding efforts for improving low-calcium-tolerance in peanut accession.

## Data availability statement

The original contributions presented in the study are included in the article/**Supplementary Material**. Further inquiries can be directed to the corresponding authors.

## Ethics statement

The author states that the peanuts involved in this study do not involve ethical relations. Experimental research on plants, including the collection of plant material, complies with relevant institutional, national, and international guidelines and legislation. Permissions were obtained to breed the cultivars or collect the sample from the bred cultivars.

## Author contributions

LL, DL, and NZ conceived and designed the study. KT carry out the test in the whole process. KT, ZL collected and curated the data. KT and ZL analyzed the data and wrote the manuscript. LL, DL, NZ and JW were responsible for funding acquisition. All authors read, revised and approved the manuscript.
